# Biomolecular Markers within the Core Axis of Aging and Particulate Air Pollution Exposure in the Elderly: A Cross-Sectional Study

**DOI:** 10.1289/ehp.1509728

**Published:** 2015-12-15

**Authors:** Nicky Pieters, Bram G. Janssen, Harrie Dewitte, Bianca Cox, Ann Cuypers, Wouter Lefebvre, Karen Smeets, Charlotte Vanpoucke, Michelle Plusquin, Tim S. Nawrot

**Affiliations:** 1 Centre for Environmental Sciences, Hasselt University, Hasselt, Belgium; 2 Primary Health Care Centre GVHV, Genk, Belgium; 3 Flemish Institute for Technological Research (VITO), Mol, Belgium; 4 Belgian Interregional Environment Agency, Brussels, Belgium; 5 Department of Public Health & Primary Care, Occupational & Environmental Medicine, Leuven University, Leuven, Belgium

## Abstract

**Background:**

Telomere length and mitochondrial DNA (mtDNA) content are markers of aging and aging-related diseases. There is inconclusive evidence concerning the mechanistic effects of airborne particulate matter (PM) exposure on biomolecular markers of aging.

**Objective:**

The present study examines the association between short- and long-term PM exposure with telomere length and mtDNA content in the elderly and investigates to what extent this association is mediated by expression of genes playing a role in the telomere–mitochondrial axis of aging.

**Methods:**

Among 166 nonsmoking elderly participants, we used qPCR to measure telomere length and mtDNA content in leukocytes and RNA from whole blood to measure expression of SIRT1, TP53, PPARGC1A, PPARGC1B, NRF1, and NFE2L2. Associations between PM exposure and markers of aging were estimated using multivariable linear regression models adjusted for sex, age, BMI, socioeconomic status, statin use, past smoking status, white blood cell count, and percentage of neutrophils. Mediation analysis was performed to explore the role of age-related markers between the association of PM exposure and outcome. Annual PM2.5 exposure was calculated for each participant’s home address using a high-resolution spatial–temporal interpolation model.

**Results:**

Annual PM2.5 concentrations ranged from 15 to 23 μg/m3. A 5-μg/m3 increment in annual PM2.5 concentration was associated with a relative decrease of 16.8% (95% CI: –26.0%, –7.4%, p = 0.0005) in telomere length and a relative decrease of 25.7% (95% CI: –35.2%, –16.2%, p < 0.0001) in mtDNA content. Assuming causality, results of the mediation analysis indicated that SIRT1 mediated 19.5% and 22.5% of the estimated effect of PM2.5 exposure on telomere length and mtDNA content, respectively.

**Conclusions:**

Our findings suggest that the estimated effects of PM2.5 exposure on the telomere–mitochondrial axis of aging may play an important role in chronic health effects of PM2.5.

**Citation:**

Pieters N, Janssen BG, Dewitte H, Cox B, Cuypers A, Lefebvre W, Smeets K, Vanpoucke C, Plusquin M, Nawrot TS. 2016. Biomolecular markers within the core axis of aging and particulate air pollution exposure in the elderly: a cross-sectional study. Environ Health Perspect 124:943–950; http://dx.doi.org/10.1289/ehp.1509728

## Introduction

Telomeres are complexes of hexameric repeats at the distal end of chromosomes where they provide stability and protection to the coding DNA. Telomere length declines with each cell division and thus can be considered as a marker of biological aging (Blackburn 1991). Excessive telomere shortening is a marker of senescence and a cause of genomic instability (Chin et al. 1999). In peripheral white blood cells, telomere shortening has been associated with age-related diseases, such as cardiovascular disease (Haycock et al. 2014) and cancer (Wentzensen et al. 2011). The natural erosion of telomeres associated with aging may be accelerated through oxidative stress and inflammation induced by environmental factors (Mitchell et al. 2014; von Zglinicki 2002). Shorter telomeres were found in peripheral white blood cells of smokers compared with nonsmokers (Nawrot et al. 2010; Valdes et al. 2005) and in persons with higher exposure to traffic-related compounds such as benzene (Hoxha et al. 2009; McCracken et al. 2010). Beside telomeres, oxidative stress also targets mitochondria (Cannino et al. 2009). Maintenance of mitochondrial function has been suggested to be an important mechanism of extending lifespan whereas decreased mitochondrial function, impaired ATP generation and increased reactive oxygen species (ROS) production are associated with aging (Sahin and DePinho 2012). Recently, Sahin et al. reported that mitochondrial changes associated with aging in telomere-deficient mice seemed to be driven by the combined suppression of peroxisome proliferator-activated receptor γ-coactivator1α *(PPARGC1A)* and peroxisome proliferator-activated receptor γ-coactivator1β (*PPARGC1B*) and their downstream targets [nuclear respiratory factor 1 (*NRF1*) and nuclear factor erythroid 2 like 2 (*NFE2L2*)] through a tumor protein p53 (*TP53*)*-*dependent repression (Sahin et al. 2011). These findings suggest a connection between the nuclear and mitochondrial aging processes (Sahin and DePinho 2012). Further evidence supporting the telomere–mitochondrial axis of aging was observed in *sirtuin1* (*SIRT1*) knock-out mice. SIRT1 belongs to a group of highly conserved NAD^+^-dependent protein deacetylases and functions as a metabolic sensor since the deacetylase activity is controlled by the cellular NAD^+^/NADH ratio (Yamamoto et al. 2007). Increased *SIRT1* expression was shown to stabilize and, in turn, increased mitochondrial biogenesis and function in cell lines (Nemoto et al. 2005) and knock-out mice (Lagouge et al. 2006).

Multiple epidemiological studies have reported associations between acute (Nawrot et al. 2011) or chronic (Brook et al. 2010; Cesaroni et al. 2014; Pope and Dockery 2006) exposure to airborne particulate matter (PM) and cardiovascular outcomes including cardiovascular morbidity and mortality. Oxidative stress and systemic inflammation have been identified as possible underlying mechanisms for effects of long-term exposure on cardiovascular outcomes (Brook et al. 2010). Since oxidative stress is linked to both telomere attrition and mitochondrial DNA (mtDNA) damage in cardiomyocytes (Moslehi et al. 2012), we hypothesized that these markers of aging may play a role in the chronic health effects of air pollution.

To date, evidence that long-term exposure to air pollution can modulate telomere length (Hou et al. 2012; Hoxha et al. 2009) and mtDNA copy number (Hou et al. 2010; Janssen et al. 2012) is limited to cross-sectional studies in healthy adults. To our knowledge, mechanisms underlying these associations have not been studied so far.

Here, we investigate whether biomolecular markers in the core axis of aging including telomere length and mitochondrial DNA are related to residential particulate air pollution exposure in elderly. Furthermore, starting from a candidate gene approach, we study possible mediators of the association between air pollution and the telomere–mitochondrial interactome. We hypothesize that the estimated effects of exposure to air pollution on mtDNA content can be mediated via telomere biology or by expression of genes in the telomere–mitochondrial axis of aging.

## Methods

The total population (*n* = 3,069) of a general medical practice in Genk, Belgium, is registered in the framework of a registration network [i.e., the integrated computerized network (INTEGO)] for family practices in Flanders, Belgium, that covers a representative part of the total Flemish population (Bartholomeeusen et al. 2005). Nonsmoking men and women, 60–80 years old, with no acute infection at enrollment and no history of malignancies, were selected in the southern region of Genk (Bijnens et al. 2013; Pennemans et al. 2011). Former smokers were only included if they stopped smoking more than 10 years before enrollment. Of those that were eligible, 166 persons were recruited by their general practitioner, which resulted in a participation rate of 92%. Due to inadequate shaking of the RNA stabilizer in the RNA sampling tube after blood draw, 41 persons (25%) lacked a suitable RNA blood sample for expression analysis. DNA samples were available for the entire study population.

Questionnaires were administered through face-to-face interviews to collect information on lifestyle, profession, education, past smoking status, age, body mass index (BMI), and gender. Family income was defined as net monthly overall family income and subdivided into low (< 1,500€), medium (1,500€–3,000€) and high (> 3,000€). Education was stratified as low (primary school), medium (high school) and high (college or university). Socioeconomic status was based on educational degree and monthly income and categorised as “low,” “middle,” and “high.” Past smokers were defined as those who had ever smoked during their life.

We gathered information on current and past use of medication from medical records of the medical practice. Reported anti-hypertensive medication included calcium antagonists, beta-blockers, alpha-blockers, and angiotensin-converting-enzyme inhibitors. We also had information whether participants used statins or not. Information on chronic obstructive pulmonary disease (COPD), asthma and myocardial infarction was obtained. After the participants had rested for 5 min, the heart rate and blood pressure were stable and seven consecutive blood pressure readings were taken by an automatic device (Stabil-O-Graph®, Köln, Germany) according to the guidelines of the European Society of Hypertension (O’Brien et al. 2003). Informed consent was obtained from all participants, and the study was approved by the Ethical Committee of the East-Limburg Hospital (ZOL) in Belgium.

### Blood Samples

Blood samples were collected in the morning until 1100 hours, after fasting in Vacutainer® Plus Plastic K2EDTA Tubes (BD, Franklin Lakes, NJ, USA) and PAXgene Blood RNA vacutainer tubes (PreAnalytiX, Qiagen, Hilden, Germany). Blood cell counts and differential leukocyte counts were determined using an automated cell counter with flow differential (Cell Dyn 3500, Abbott Diagnostics, Abott Park, IL, USA). Blood glucose levels, total cholesterol, high-density lipoprotein (HDL) cholesterol, low-density lipoprotein (LDL) cholesterol, triglycerides, and C-reactive protein (CRP) were measured according to standard clinical procedures.

### DNA Analysis

Total DNA was extracted from white blood cells of the buffy coat using the MagMAX™ DNA Multi-Sample kit (Applied Biosystems, Foster City, CA, USA) following the manufacturer’s instructions. The yield (ng/μL) and purity ratios (A260/280 and A260/230) of the extracted DNA was determined with the NanoDrop spectrophotometer (ND-1000, Isogen Life Science, De Meern, Netherlands). Extracted DNA was stored at –20°C until further use.

### Measurement of Leukocyte Mitochondrial DNA Content

Relative mtDNA content was determined using a quantitative real-time PCR (qPCR) assay by taking the ratio of two mitochondrial gene copy numbers (*MTF3212/R3319* and mitochondrial encoded NADH dehydrogenase [*MT-ND1*]) to two single-copy nuclear reference genes (acidic ribosomal phosphoprotein P0 [*RPLP0*] and beta actin [*ACTB*]) (Pieters et al. 2015). The forward and reverse primers for the mitochondrial genes were respectively 5´-CACCC​AAGAA​CAGGG​TTTGT-3´ and 5´-TGGCC​ATGGG​TATGT​TGTTA​A-3´ for *MTF32​12/33​19*, and 5´-ATGGC​CAACC​TCCTA​CTCCT-3´ and 5´-CTACA​ACGTT​GGGGC​CTTT-3´ for *MT-ND1*. For the reference genes, the forward and reverse primers were respectively 5´-ACTCT​TCCAG​CCTTC​CTTCC-3´ and 5´-GGCAG​GACTT​AGCTT​CCACA-3´ for *ACTB*, and 5´-GGAAT​GTGGG​CTTTG​TGTTC-3´ and 5´-CCCAA​TTGTC​CCCTT​ACCTT-3´ for *RPLP0*. Each sample was run in duplicate for the nuclear genes and in triplicate for the mitochondrial genes. A 10 μL PCR reaction medium contained Fast SYBR® Green I dye 2× (Applied Biosystems, Lennik, Belgium) mastermix, forward (300 nM) and reverse (300 nM) primer, and 12.5 ng DNA. All PCR-reactions were performed on a 7900HT Fast Real-Time PCR System (Applied Biosystems, Foster City, CA, USA). The thermal cycling profile was similar for mtDNA and nuclear DNA: 20 sec at 95°C to activate the AmpliTaq Gold® DNA-polymerase, followed by 40 cycles of 1 sec at 95°C for denaturation, and 20 sec at 60°C for annealing/extension. Each run was completed by a melting curve analysis to confirm the amplification specificity and absence of non-specific PCR products. Each PCR plate contained six inter-run calibrators (IRCs) and two no-template controls (NTCs). After thermal cycling, raw data were collected and processed. Cq values of the mitochondrial genes were normalized relative to the two reference genes using the qBase software (Biogazelle, Zwijnaarde, Belgium). The program uses modified software from the classic comparative delta-delta-Ct method that takes into account multiple reference genes and uses inter-run calibration algorithms to correct for run-to-run differences (Hellemans et al. 2007). Coefficient of variation (CV) within triplicates was 1.7% for mitochondrial genes and 1.6% for duplicates for the reference genes.

### Measurement of Leukocyte Telomere Length

Telomere length was measured as telomere repeat copy number relative to two single gene copy numbers (T/S ratio) by a modified version of the previously described PCR-based telomere assay by Cawthon (Cawthon 2009; Pieters et al. 2015). The forward and reverse primer for the telomeres were 5´-ACACT​AAGGT​TTGGG​TTTGG​GTTT​GGGTT​TGGGT​TAGTGT-3´ and 5´-TGTTA​GGTAT​CCCTA​TCCCT​ATCCC​TATCC​CTATC​CCTAA​CA-3´. The primers of the reference genes (*ACTB* and *RPLP0*) were the same as used for the mtDNA content measurement. The telomere reaction medium contained Fast SYBR® Green I dye 2× (Applied Biosystems, Lennik, Belgium) mastermix, forward (100 nM) and reverse (900 nM) primer, and 12.5 ng DNA. The telomere reactions were performed in triplicate. The thermal cycling profile for the telomere reaction consisted of the following steps: 20 sec at 95°C, 2 cycles of 15 sec at 94°C and 15 sec at 49°C, and 40 cycles of 15 sec at 94°C, 10 sec at 62°C, and 15 sec at 74°C. Amplification specificity and absence of primer dimers was confirmed by melting curve analysis at the end of each run. Each PCR-plate contained six IRCs and two NTCs. We also included two control samples, one with relatively short telomeres and one with relatively long telomeres. Cq values of the telomere assay were normalized to two reference genes while taking into account run-to-run differences using qBase software (Biogazelle, Zwijnaarde, Belgium). CV within triplicates was 2.6% for telomeres and 1.6% for duplicates for the reference genes. CV for the exponentiated T/S ratio was less than 7%.

### Gene Expression Analysis

Total RNA was extracted from PAXgene Blood RNA vacutainer tubes (PreAnalytiX, Qiagen, Hilden, Germany) with the PAXgene Blood RNA kit (PreAnalytiX) according to the manufacturer’s instructions. For 41 persons no suitable RNA sample could be collected. cDNA was synthesized from 500 ng RNA using the GoScript™ Reverse Transcription System (Promega, Madison, WI, USA) according to the manufacturer’s instructions. A qPCR reaction was set up by adding 6.6 ng cDNA together with 5 μL Taqman® Fast Advanced Master Mix (Life Technologies, Foster City, CA, USA) and 0.5 μL PrimeTimeTM assay (Integrated DNA Technologies, Coralville, IA, USA) in a final reaction volume of 10 μL. Cycling conditions for all transcripts were 2 min at 50°C, 10 min at 95°C and 40 cycles of 15 sec at 95°C, and 1 min at 60°C. Overall, we studied the gene expression of candidate genes within the telomere*-TP53*-*PPARGC1A*-mitochondrial axis of aging (Lee and Wei 2005; Sahin and DePinho 2012) (Table 1). Each qPCR reaction was carried out in triplicate and three NTCs and six IRCs were included in each 384-well plate. Amplification efficiencies of PrimeTime assays were determined by standard dilution series of a mixed sample, resulting in an efficiency between 90% and 110% for all assays and the amplification specificity was confirmed by visualization of the expected band size on a 4% agarose gel. After thermal cycling, Cq values were collected and normalized to three reference genes, taking into account run-to-run differences using IRCs with qBase software (Biogazelle, Zwijnaarde, Belgium). Tyrosine 3-monooxygenase/tryptophan 5-monooxygenase activation protein, zeta polypeptide (*YWHAZ*), hypoxanthine phosphoribosyltransferase 1 *(HPRT1*), and *RPLP0* were selected via geNorm and Normfinder as reference genes to normalize the data.

**Table 1:** Assay information of the selected candidate genes.

Abbreviation	IDT assay	Gene name	Ref seq number	Primer efficiency (%)^*a*^	Exon location	Amplicon length (bp)
*HPRT1*	Hs.PT.39a.22214821	Hypoxanthine phosphoribosyltransferase 1	NM_000194	93	6–8	128
*NRF1*	Hs.PT.56a.3666627	Nuclear respiratory factor 1	NM_005011	106	12–13	102
*NFE2L2*	Hs.PT.56a.40946676.gs	Nuclear factor erythroid 2-like 2	NM_006164	99	4–5	124
*PPARGC1A*	Hs.PT.56a.40982761	Peroxisome proliferator-activated receptor γ, coactivator 1 α	NM_013261	99	12–13	133
*PPARGC1B*	Hs.PT.56a.38577994	Peroxisome proliferator-activated receptor γ, coactivator 1 β	NM_133263	105	12–13	102
*RPLPO*	Hs.PT.56a.40434846	Acidic ribosomal phosphoprotein P0	NM_053275	101	7–8	146
*SIRT1*	Hs.PT.56a.40870995	Sirtuin 1	NM_001142498	94	9–10	133
*TP53*	Hs.PT.56a.39489752.g	Tumor protein p53	NM_001126114	90	16–16	146
YWHAZ	Hs.PT.39a.22214858	Tyrosine 3-monooxygenase/tryptophan 5-monooxygenase activation protein, zeta polypeptide	NM_003406	106	1–2	135
bp, base pairs; IDT, integrated DNA technologies. ^***a***^Primer efficiency was calculated by a standard dilution series and using the formula: efficiency = 10^(–1/slope)^ – 1.						

### Exposure Measurement

The annual exposure levels of PM_2.5_ were estimated for each participant’s home address using a high-resolution spatial interpolation method (kriging method) (Janssen et al. 2008) that uses pollution data collected in the official fixed-site monitoring network and land cover data obtained from satellite images (CORINE land-cover data set) in combination with a dispersion model (Lefebvre et al. 2011, 2013). The dispersion model described by Lefebvre et al. (2011, 2013) uses the results from the interpolation method as background and superimposes the effects of industrial point sources and line sources from traffic to calculate the concentrations on a predefined grid. A correction for double counting was applied. This model chain provides daily PM_2.5_ values that are aggregated to annual (1 year), past month (30 days), and past week (7 days) means for each participant’s home address. Annual means were considered as a proxy of long-term exposure to PM_2.5_. Validation statistics of the interpolation tool gave a temporal explained variance (*R* ^2^) for hourly averages of 0.88 and spatial *R* ^2^ for annual mean PM_2.5_ of 0.83 (Maiheu et al. 2012).

### Statistical Analysis

Statistical analyses were conducted using the SAS statistical package, version 9.3 (SAS Institute, Cary, NC, USA). Gene expression data, telomere length, and mtDNA content were log_10_-transformed to better approximate a normal distribution. We used regression models to study the association between long-term (annual) and short-term (last month and last week) exposure to particulate air pollution and aging-related markers. Additionally, we built a multiple exposure model where we fitted all three exposure windows in the same regression model. We considered the following *a priori* chosen model covariates: sex, age (years), BMI (kg/m^2^), socioeconomic status (low/middle/high), statin use (yes/no), past smoking status (yes/no), white blood cell count, and percentage of neutrophils. Pearson correlation coefficients were calculated between leukocyte telomere length, mtDNA content, the studied candidate genes, and annual PM_2.5_ exposure to determine which genes to evaluate in a mediation analysis.

Formal mediation analysis was performed to explore the role of *SIRT1* and telomere length as mediators of the association between exposure to particulate air pollution and markers of aging. This approach decomposes the total observed effect of exposure on markers of aging into a direct effect and an indirect effect that acts via the mediator of interest (Valeri and Vanderweele 2013). We only analyzed intermediates that satisfied all the assumptions of mediation analysis (i.e., a significant relation of the outcome to the exposure, a significant relation of the outcome to the mediator, and a significant relation of the mediator to the exposure) as potential mediators (e.g., *SIRT1* and telomere length). However, these estimates should be interpreted with caution because the underlying assumptions of causality, as in all observational studies, between each pair of factors in the analysis cannot be verified.

In a sensitivity analysis, we explored whether following continuous covariates such as glucose, HDL, LDL, CRP, and systolic and diastolic blood pressure may alter the association between our biomolecular markers of aging and long-term PM_2.5_ exposure. Furthermore, we performed an analysis excluding participants who lived at their home address for < 10 years. A *p*-value of < 0.05 was considered to be statistically significant.

## Results

### Study Population Characteristics

Descriptive characteristics of the study population are displayed in Table 2. Overall, the study population consisted of 166 elderly participants with a mean age (± SD) of 70.6 ± 4.7 years. BMI (± SD) averaged 27.5 ± 3.7 kg/m^2^ and 27.3 ± 5.1 kg/m^2^ for men and women, respectively. Of the 166 elderly participants, 89 (54%) were former smokers. The majority of former smokers were men (76%). Mean (± SD) pack-years for former smokers was 19.0 ± 17.7 and 13.3 ± 12.5 for men and women, respectively. The average annual mean (± SD) PM_2.5_ concentration at the residence of study participants was 21.1 ± 1.76 μg/m^3^ (range 15.5 μg/m^3^–23.4 μg/m^3^). The average duration of living at the residential address was 37.6 ± 16.9 years (5–95th percentile: 9–73 years).

**Table 2:** Characteristics of the study population, stratified by sex.

Characteristics	Men (*n *= 77)	Women (*n *= 89)
Age (years)	70.2 ± 5.1	70.8 ± 4.3
BMI (kg/m^2^)	27.5 ± 3.7	27.3 ± 5.1
Former smoker	58 (76%)	31 (35%)
Pack years	19.0 ± 17.7	13.3 ± 12.5
Socioeconomic status^*a*^		
Low	28 (37%)	37 (42%)
Middle	35 (45%)	34 (38%)
High	14 (18%)	18 (20%)
Statin use	45 (58%)	41 (46%)
HDL cholesterol (mg/dL)	53.4 ± 18.2	66.3 ± 17.5
LDL cholesterol (mg/dL)*	112.9 ± 31.6	119.1 ± 36.3
High sensitivity C reactive protein (mg/dL)	0.12 ± 0.3	0.13 ± 0.3
Glucose (mg/dL)	106.1 ± 37.9	100 ± 29.5
Myocardial infarction	8 (10%)	6 (7%)
COPD*	10 (13%)	2 (2%)
Asthma	4 (5%)	2 (2%)
Systolic blood pressure	147.3 ± 18.2	142 ± 19.4
Diastolic blood pressure	90.6 ± 12.6	84.4 ± 11.6
Telomere length^*b*^	1.04 ± 0.05	1.04 ± 0.04
mtDNA content^*b*^	0.71 ± 0.04	0.78 ± 0.04
Average annual PM_2.5 _(μg/m^3^)	21.1 ± 1.72	21.1 ± 1.75
Time at current residential address (years)	35.9 ± 17.0	39.3 ± 16.8
^***a***^Socioeconomic status was based on educational degree and monthly family income. ^***b***^Geometric mean ± SD. *p < 0.05 between men and women.		

### Association Between Air Pollution Indicators and Markers of Aging

Based on a simple linear regression model, telomere length (T/S ratio) was 4.06% lower [95% confidence interval (CI): –8.06%, 0.61%; *p* = 0.09] in association with a 1-year increase in age in the study population (age range 60–80 years), while mtDNA content was not associated with age (–0.62%, 95% CI: –2.07%, 0.88%, *p* = 0.41). Of the selected candidate genes, only *SIRT1* expression was significantly correlated with telomere length, mtDNA content, and annual PM_2.5_ exposure (Table 3).

**Table 3:** Pearson correlation matrix between leukocyte telomere length, mtDNA content, the studied candidate genes, and annual PM_2.5_ exposure.

	Telomere length	mtDNA content	*SIRT1*	*PPARGC1A*	*NRF1 *	*TP53*	*PPARGC1B*	*NFE2L2*
mtDNA content	0.22**
*SIRT1*	0.27**	0.43***
*PPARGC1A*	0.05	0.04	0.20*
*NRF1*	0.22*	0.26**	0.74***	0.22*
*TP53*	–0.20*	0.02	–0.11	0.12	0.03
*PPARGC1B*	–0.06	–0.01	0.07	0.20*	0.42***	0.40***
*NFE2L2*	–0.001	0.02	–0.05	0.07	0.03	0.44***	0.24**
Annual PM_2.5_ exposure	–0.29***	–0.40***	–0.26**	0.03	–0.03	0.15	0.03	0.04
*p < 0.05; **p < 0.01; ***p < 0.001.								

**Figure 1:**
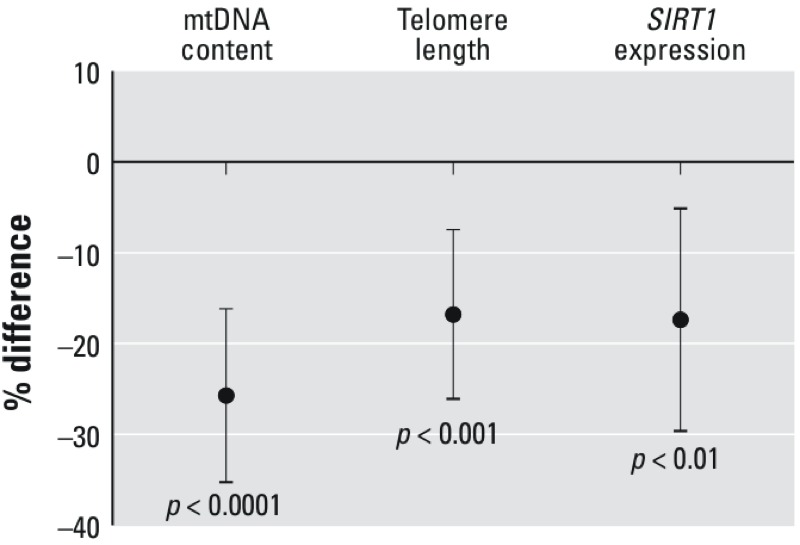
Relative percentage (%) difference in mitochondrial DNA content (*n *= 166), telomere length (T/S) ratio (*n *= 166), and *SIRT1* expression (*n *= 125) in association with a 5-μg/m^3^ increment in average annual PM_2.5_ exposure adjusted for sex, age, BMI, socioeconomic status (based on education and monthly family income), statin use, past smoking status, white blood cell count, and percentage of neutrophils.

Biomolecular markers of aging (mtDNA content, telomere length, and *SIRT1* expression) were inversely associated with long-term PM_2.5_ exposure (Table 3). In multivariate models adjusted for sex, age, BMI, socioeconomic status, statin use, past smoking status, white blood cell count and percentage of neutrophils, a 5-μg/m^3^ increment in average annual PM_2.5_ exposure was associated with a 16.8% (95% CI: –26.0%, –7.4%, *p* = 0.0005) relative decrease in telomere length, a 25.7% (95% CI: –35.2%, –16.2%, *p* < 0.0001) relative decrease in mtDNA content, and a 17.3% (95% CI: –30.0%, –5.1%, *p* = 0.006) relative decrease in *SIRT1* expression (Figure 1).

In contrast with the negative associations between annual average PM_2.5_ exposure and telomere length, mtDNA content, and *SIRT1* expression, monthly average PM_2.5_ exposures were associated with longer telomeres, greater mtDNA content, and higher *SIRT1* expression (Table 4). Average PM_2.5_ exposures in the previous week was also positively associated with mtDNA content and *SIRT1* expression but was not associated with telomere length (Table 4). Furthermore, we fitted all three exposure windows as independent variables in the same regression model but this did not change our findings (Table 4).

**Table 4:** Associations between an IQR increase in average PM_2.5_ exposure during the previous year (long-term exposure) or during the previous month or week (short-term exposure) and log_10_-transformed telomere length (T/S ratio), mtDNA content, and *SIRT1* expression.

Exposure model^*a*^	Telomere length		mtDNA content		*SIRT1* expression	
	Estimated effect (95% CI)	*p*-Value	Estimated effect (95% CI)	*p*-Value	Estimated effect (95% CI)	*p*-Value
Single exposure^*b*^
Last year	–0.040 (–0.065, 0.017)	0.0005	–0.065 (–0.094, –0.038)	0.0001	–0.041 (–0.077, –0.011)	0.006
Last month	0.029 (0.017, 0.041)	0.0001	0.038 (0.025, 0.050)	0.0001	0.033 (0.015, 0.052)	0.0005
Last week	0.003 (–0.007, 0.014)	0.5	0.029 (0.09, 0.039)	0.001	0.034 (0.016, 0.051)	0.0003
Multiple exposure^*c*^
Last year	–0.032 (–0.053, –0.011)	0.0031	–0.055 (–0.074, –0.035)	0.0001	–0.039 (–0.066, –0.012)	0.005
Last month	0.028 (0.015, 0.040)	0.0001	0.023 (0.012, 0.04)	0.0001	0.022 (0.002, 0.041)	0.03
Last week	–0.004 (–0.014, 0.006)	0.4	0.022 (0.013, 0.032)	0.0001	0.024 (0.005, 0.043)	0.01
IQR, interquartile range. ^***a***^The IQR was 2.50 μg/m^3 ^for past-year exposure, 2.14 μg/m^3 ^for last-month exposure, and 3.37 μg/m^3 ^for last-week exposure. ^***b***^Single exposure models were adjusted for sex, age, BMI, socioeconomic status (based on education and monthly family income), statin use, past smoking status, white blood cell count, and percentage of neutrophils. ^***c***^In the multiple exposure model, all three exposure windows were fitted in the same regression model.						

### Mediation Analysis

**Figure 2:**
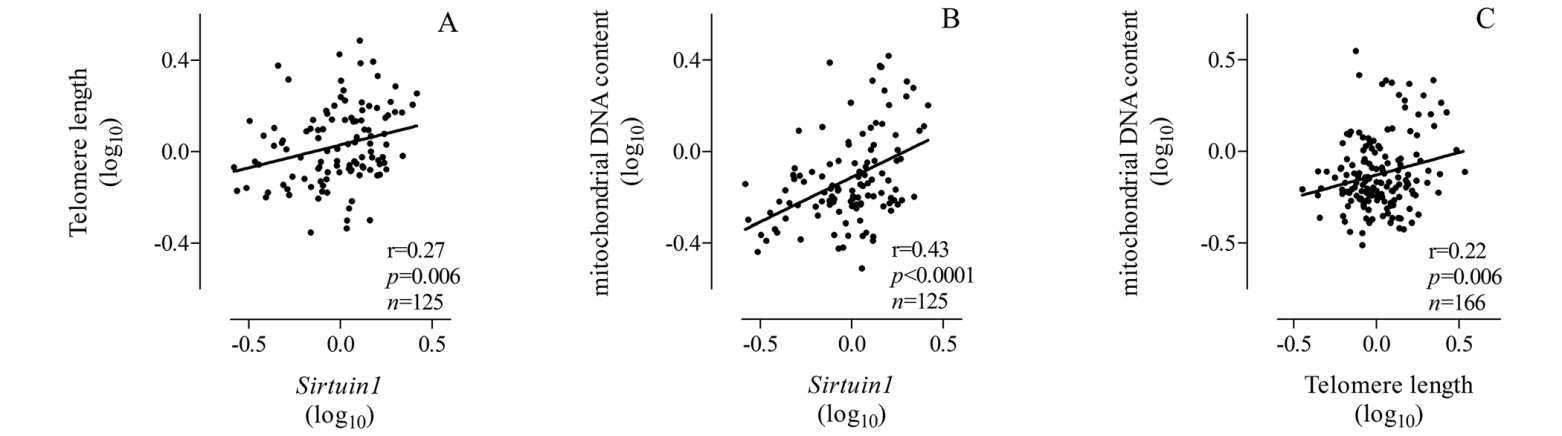
Correlation between (*A*) telomere length and *SIRT1* expression, (*B*) mtDNA content and *SIRT1* expression, and (*C*) mtDNA content and telomere length.

We performed mediation analysis to estimate the proportion of the associations between PM_2.5_ exposure and leukocyte mtDNA content that might be mediated by telomere length or *SIRT1* expression if underlying causal assumptions of the mediation analysis are valid. We selected *SIRT1* for evaluation as a potential mediator because it was significantly associated with PM_2.5_ exposure (Figure 1) as well as with mtDNA content and telomere length (Figure 2), in contrast with the other candidate genes, which did not meet this criterion. In addition, we also evaluated telomere length as a potential mediator of the association between PM_2.5_ exposure and mtDNA content.

**Figure 3:**
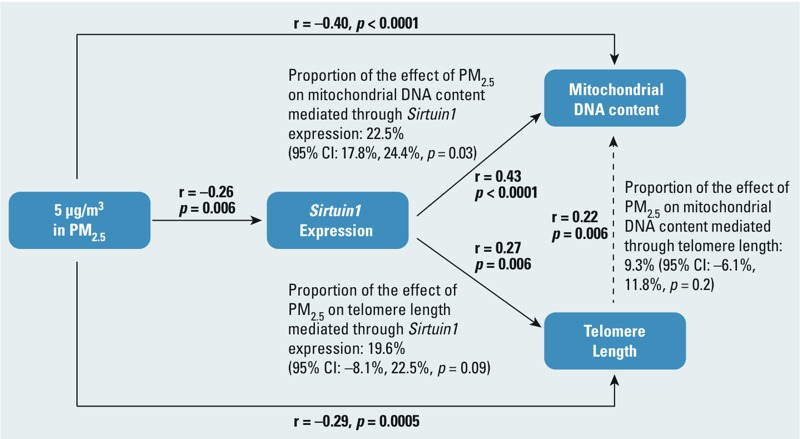
Mediation analysis showing the estimated proportion of associations between PM_2.5_ (μg/m^3^) exposure and leukocyte mtDNA content and telomere length, mediated through *SIRT1* expression if underlying assumptions of the analysis are valid.

Assuming that requirements for the mediation analysis were valid, we estimated that *SIRT1* expression mediated 19.6% (95% CI: –8.1%, 22.5%, *p* = 0.09) of the inverse association between PM_2.5_ exposure and telomere length, and 22.5% (95% CI: 17.8%, 24.4%, *p* = 0.03) of the inverse association between PM_2.5_ exposure and mtDNA content (Figure 3). The analysis did not indicate statistically significant mediation of the inverse association between PM_2.5_ exposure and mitochondrial DNA content by telomere length (Figure 3).

### Sensitivity Analysis

To test the robustness of our results, we determined the influence of different metabolic parameters on the association between our biomolecular markers of aging and long-term PM_2.5_ exposure. In separate models, we adjusted the main model for glucose, HDL, LDL, CRP, and systolic and diastolic blood pressure. Adjustment for the additional covariates did not show strong confounding and did not substantially change estimates between the main model (Table 5). To further test the robustness of our results, we added an analysis excluding participants who lived at their home address for < 10 years (*n* = 10). This did not alter the reported associations (Table 5).

**Table 5:** Sensitivity analysis of the association between an IQR increase for past-year PM_2.5_ exposure (2.5 μg/m^3^) in annual average PM_2.5_ exposure and leukocyte log_10_-transformed telomere length (T/S ratio) and mtDNA content.

Model variable	Telomere length		mtDNA content	
	Estimated effect (95% CI)	*p*-Value	Estimated effect (95% CI)	*p*-Value
Main model	–0.040 (–0.065, –0.017)	0.0005	–0.065 (–0.094, –0.038)	0.0001
+ Glucose	–0.039 (–0.61, –0.017)	0.0006	–0.059 (–0.082, –0.037)	0.0001
+ CRP	–0.034 (–0.057, –0.011)	0.003	–0.056 (–0.078, –0.034)	0.0001
+ HDL	–0.032 (–0.054, –0.009)	0.0057	–0.057 (–0.080, –0.035)	0.0001
+ LDL	–0.034 (–0.057, –0.011)	0.0036	–0.061 (–0.084, –0.038)	0.0001
+ Systolic BP	–0.037 (–0.060, –0.014)	0.0022	–0.061 (–0.085, –0.037)	0.0001
+ Diastolic BP	–0.037 (–0.061, –0.14)	0.0022	–0.061 (–0.085,–0.037)	0.0001
Excluding participants < 10 years at current address	–0.043 (–0.065, –0.021)	0.0002	–0.064 (–0.088, –0.041)	0.0001
The main model, adjusted for sex, age, BMI, socioeconomic status (based on education and monthly family income), statin use, past smoking status, white blood cell count, and percentage of neutrophils, was additionally adjusted for each listed covariate in a separate model. A separate sensitivity analysis was performed using the main model while excluding participants that lived < 10 years at their current address.				

## Discussion

We demonstrated that molecular targets in the core axis of aging in the elderly are influenced by residential particulate air pollution. The key finding of our study is that residential annual average PM_2.5_ exposure was associated with lower mtDNA content, shorter telomere length, and reduced *SIRT1* expression in peripheral blood leukocytes of the elderly. If underlying causal assumptions of the mediation analysis are valid, *SIRT1* expression mediates a significant proportion of the association between annual average PM_2.5_ exposure and lower mtDNA content on telomere length.

Among the 166 nonsmoking elderly participants, telomere length was associated with a 4.06% decrease per annual increase of age, within the age range of 60–80 years. We found that a 5-μg/m^3^ increment in annual PM_2.5_ was associated with a 16.8% decrease in telomere length and a 25.7% decrease in mtDNA content while adjusting for sex, age, BMI, socioeconomic status, and statin use. The public health significance of the association between annual average PM_2.5_ exposure and telomere length in our population can be illustrated by the fact that it corresponds to the estimated decrease in T/S ratio associated with a 4-year increase in age in our study population. These associations were estimated for a study population with estimated average annual exposure of 21.1 μg/m^3^ (range 15.5–23.4 μg/m^3^), which are below the annual average PM_2.5_ limits for the EU (25 μg/m^3^) but above the U.S. PM_2.5_ limit (12 μg/m^3^) (Esworthy 2015).

Extensive epidemiological studies support the associations between ambient air pollution and adverse health outcomes, including cardiovascular and respiratory disease, both with short-term (Levy et al. 2000; Nawrot et al. 2011) and chronic exposure (Beelen et al. 2014a, 2014b; Hamra et al. 2014; Laden et al. 2006). To date, studies examining the associations between PM exposure and telomere length reported different telomere responses after long-term or short-term exposure to PM. Short-term metal-rich PM exposure was positively associated with leukocyte telomere length in 63 steel workers (Dioni et al. 2011) and in a study of 120 office workers and 120 truck drivers in Beijing, China (Hou et al. 2012), and an IQR increase in annual black carbon exposure (0.25 μg/m^3^) was associated with an 8% decrease in leukocyte telomere length (95% CI: –13%, –2%) in a cohort of 165 never-smoking elderly men (McCracken et al. 2010). Consistent with these studies, we found that short-term (last month) exposure to PM_2.5_ was associated with increased telomere length, whereas long-term (annual) exposure to PM_2.5_, with subsequent cumulative burden of oxidative stress and inflammation, was associated with shorter telomere length. Whether acute increases in telomere length are due to telomerase activation, effects on telomere associated proteins, or clonal expansion of less mature leukocytes needs to be evaluated (Hodes et al. 2002; Weng et al. 1997). Similar differences between short-term and chronic exposure to PM were also reported in association with mtDNA content. Short-term exposure to PM was positively associated with mtDNA content in the steelworkers study (Hou et al. 2010) but negatively associated with mtDNA content in the study of Beijing office workers and truck drivers (Hou et al. 2013). A study performed in 178 newborns from the ENVIR*ON*AGE birth cohort in Belgium (Janssen et al. 2012) revealed that a 10-μg/m^3^ increment in PM_10_ exposure during the last trimester of pregnancy was associated with a 17.4% (95% CI: –31.8%, –0.1%) decrease in placental mtDNA content.

The biological mechanisms by which air pollution may cause adverse health outcomes are not completely understood, but oxidative stress and inflammation are thought to be of importance. The ability of oxidative stress to damage nucleic acids provides a potential mechanism by which it could interfere with telomere DNA (Epel et al. 2004). Due to their high content of guanine, telomeres are highly sensitive to ROS-induced damage (Grahame and Schlesinger 2012). Furthermore, single strand DNA breaks in telomeric DNA are not repaired efficiently (Kruk et al. 1995). In addition to direct effects to DNA, telomere attrition results from somatic cell replication. Oxidative stress and inflammation promote this process. Telomere length represents a record of the replicative history of cells and might be an index of cumulative oxidative stress (von Zglinicki and Martin-Ruiz 2005). Accelerated shortening of telomeres, and as such, senescence of cells may be an important pathway by which oxidative stress may accelerate biological aging and the resultant development of aging-related morbidity, including cardiovascular disease.

A recent experimental study in knock-out mice (Sahin et al. 2011) and cross-sectional studies in humans (Kim et al. 2013; Pieters et al. 2015; Qiu et al. 2015) provide evidence of the relationship between mtDNA content and telomere length and form a mechanistic platform for age-related disease (Moslehi et al. 2012). Our study is the first to report the intermediate mechanisms of PM-induced mtDNA alterations by investigating the role of telomere length and *SIRT1* expression. We showed that the association between PM_2.5_ and mtDNA content might be mediated by *SIRT1* expression. A study in yeast shows that SIRT1 suppression increases telomerase activity (Palacios et al. 2010) and also inactivates the “guardian of the genome,” *TP53* (Vaziri et al. 2001). In addition, *SIRT1* activates *PPARGC1A*, a regulator of mitochondrial biogenesis (Aquilano et al. 2010). Overexpression of *SIRT1* in mice strains was shown to reduce incidence of several age-related diseases such as cardiovascular disease, metabolic disease, and cancer (Donmez and Guarente 2010). However, we only found significant associations between air pollution and *SIRT1* expression with two biomolecular markers of aging (telomere length and mtDNA content). However, the correlation of the other candidate genes was as expected with the biomolecular markers of aging; for example, *TP53* correlates inversely with telomere length and *SIRT1* expression. *SIRT1* expression was also positively correlated with *PPARGC1A*, as expected from the literature (Sahin and DePinho 2010).

Some limitations of this study warrant consideration. Although the results were consistent after multiple adjustments, we cannot exclude that our findings were caused by some unknown factor that is associated with both mitochondrial function and telomere length. Although we used recently developed statistical methods on causal interference (Valeri and Vanderweele 2013), these methods can never prove the biological direction (causality) of the findings and estimates should be interpreted with considerable caution. Secondly, telomere length, mtDNA content, and gene expression were measured in a mixture of cells in which composition differences between samples could influence our associations. In addition, changes in mtDNA content in human blood cells could also be attributed to platelet variation (Cozzarizza et al. 2003). Platelet contamination increases mtDNA without an augmentation in nuclear DNA and affects mtDNA content (Urata et al. 2008). However, in a previous study by Janssen et al. (2012), mtDNA did not correlate with blood platelets, neutrophils, white blood cells or white blood cell/platelet ratio. Nevertheless, we adjusted all our analysis for white blood cell count and percentage of neutrophils.

## Conclusion

In our study population of elderly non-smokers, we showed that annual average PM_2.5_ exposure was inversely associated with telomere length, mtDNA content, and *SIRT1* expression. These observations provide additional evidence that may be relevant for the mechanism of action of air pollution. To our knowledge, this is the first study investigating the potential influence of the telomere–mitochondrial core axis of aging on associations between air pollution and health effects. The public health significance of the association between PM_2.5_ exposure and telomere length in our population is consistent with the estimated decrease in T/S ratio associated with a 4-year increase in age in our study population.

***Editor’s Note:** In the Advance Publication, the titles of Tables 4 and 5 incorrectly indicate that estimated effects were for a 5-μg/m^3^ increase in average PM_2.5_, rather than an IQR increase. The IQR for past-year exposure is 2.50 μg/m^3^, for last-month exposure 2.14 μg/m^3^, and for last-week exposure 3.37 μg/m^3^. Furthermore, in Table 4 the 95% CI of *SIRT1* expression for last-year exposure should be (−0.077, −0.011). In addition, values given in the second paragraph of the “Discussion” for the range and annual average PM_2.5_ exposure estimates in the study population are incorrectly listed as 20.8 μg/m^3^ (range 15.7–23.0 μg/m^3^); the correct values are 21.1 μg/m^3^ (range 15.5–23.4 μg/m^3^). The corrections are included in this article. The authors regret these errors.*
